# Molecular Refinement of Clinical Staging in Hepatocellular Carcinoma Patients Evaluated for Potentially Curative Therapies

**DOI:** 10.1371/journal.pone.0023093

**Published:** 2011-09-01

**Authors:** Alessandro Vitale, Filippo Navaglia, Rafael Ramírez Morales, Anna Chiara Frigo, Daniela Basso, Francesco D'Amico, Giacomo Zanus, Pasquale Bonsignore, Fabio Farinati, Patrizia Burra, Marco Senzolo, Francesco Grigoletto, Mario Plebani, Umberto Cillo

**Affiliations:** 1 Unità di Chirurgia Epatobiliare e Trapianto Epatico, Dipartimento di Chirurgia Generale e Trapianti d'Organo, Università di Padova, Padova, Italy; 2 Dipartimento di Medicina di Laboratorio, Università di Padova, Padova, Italy; 3 Istituto Oncologico Veneto IOV – IRCCS, Padova, Italy; 4 Unità di Biostatistica ed Epidemiologia, Dipartimento di Medicina Ambientale e Sanità Pubblica, Università di Padova, Padova, Italy; 5 Divisione di Gastroenterologia, Dipartimento di Scienze Chirurgiche e Gastroenterologiche, Università di Padova, Padova, Italy; University of Texas Southwestern Medical Center at Dallas, United States of America

## Abstract

**Aim:**

VEGF and AFP mRNA determinations in the blood are promising prognostic factors for patients with HCC. This study explores their potential prognostic synergy in a cohort of HCC patients evaluated for potentially curative therapies.

**Methods:**

One hundred twenty-four patients with a diagnosis of HCC were prospectively enrolled in the study. Inclusion criteria were: (a) histological diagnosis of HCC and assessment of tumour grade and (b) determination of AFP mRNA status and VEGF levels in the blood before therapy.

**Results:**

At baseline evaluation, 40% of the study group had AFP mRNA in the blood (AFP mRNA positive), and 35% had VEGF
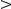
23 pg ml^−1^ (VEGF positive). Surgery was performed in 58 patients (47%), 54 (43%) had tumour ablation, and 12 had chemoembolisation (10%). Median follow-up and survival of the study group were 19 and 26 months (range, 1 to 60), respectively. The association of AFP mRNA and VEGF proved to be prognostically more accurate than their single use in discriminating the risk of death (ROC curve analysis) and survival probability (Cox analysis). In particular, we identified 3 main molecular stages (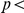
0,0001): both negative (3-year survival = 63%), one positive (3-year survival = 40%), both positive (3-year survival = 16%). Multivariate analysis identified BCLC staging, surgery, and molecular staging as the most significant survival variables.

**Conclusions:**

The preoperative determination of AFP mRNA status and VEGF may potentially refine the prognostic evaluation of HCC patients and improve the selection process for potentially curative therapies.

## Introduction

The biological history of HCC is closely related to the process of angiogenesis in neoplastic nodules [1]. Angiogenesis plays an important role in cancer from the initial stage of carcinogenesis to the end stage of metastatic disease: first, blood vessels provide a route for supply of nutrients and oxygen to sustain tumour growth; second, neovessels provide access for tumour cells to enter the circulation [2].

During this complex process, a consensual de-differentiation of HCC and endothelial cells is of paramount importance in determining tumour aggressiveness as defined by the risk of vascular invasion and intra-extra hepatic metastasis. This physio-pathological model explains the crucial prognostic role of tumour grade and mVI as predictors of overall and disease-free survival in surgical and non-surgical series [3], [4].

In current clinical practise, however, there are no accurate preoperative diagnostic tools available for tumour grade and mVI [5], such that only tumour size and number are currently used to stage HCC patients when determining the therapeutic approach [6]. This explains why, in currently used HCC staging systems, there is a substantial variation in prognosis among patients within the same stage [7].

In this context, circulating molecular markers have the potential to be used as simple and effective tools to refine the prognostic prediction and treatment of HCC patients. Among the proposed circulating molecular markers [8], serum VEGF level [9] and the presence of AFP mRNA in the blood [10], [11] show promise for patient prognosis.

VEGF is the best known angiogenic factor produced by tumours (it has mitogenic effects on endothelial cells and promotes vascular permeability), but recent studies [1] have also shown a direct autocrine role on HCC growth and aggressiveness (mitogenic effects on tumour cells, promotion of HCC de-differentiation, disruption of HCC cell tight junctions).

AFP mRNA is a specific marker of HCC cells in the circulation and several studies have shown a correlation with HCC-aggressive tumour features (nodule size, number, vascular invasion, grading) [10], [12]. It may therefore be considered an indirect marker of tumour growth and de-differentiation, angiogenesis (vascular permeability and invasion) and micro-metastasis in advanced cases.

The controversial prognostic accuracy of these biomarkers used alone, however, has not yet justified their introduction into clinical practise. We hypothesized that the combination of the most significant angiogenic factor (VEGF) and the most significant circulating HCC cell marker (AFP mRNA) could be used to improve the overall accuracy of HCC prognostic prediction and treatment decisions.

On this basis, we started a prospective observational study for preoperative determination of VEGF serum levels and AFP mRNA in a cohort of HCC patients referred to our tertiary level surgically oriented unit in order to evaluate the indication for prognosis prediction in potentially curative therapies.

## Results

### Patient characteristics

In the study period, 124 HCC patients were enrolled. Baseline patient characteristics are described in Table 1. Median age was 62years (13 to 88), and the male/female ratio was 98/26. All patients had cirrhotic livers, and 59 (48%) had impaired liver function (Child-Pugh B–C). The most common ætiology was HCV. CRPH was diagnosed in 89 patients (78%), whereas general condition was deteriorated (PST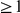) in 22 patients (18%).

**Table 1:** Baseline characteristics of the enrolled patients.

Variables	Patients	(124)
**Median age (years)**	62	(13–88)
**Sex (males)**	98	(80%)
**Cirrhosis ætiology**		
HCV	65	(52%)
HBV	24	(20%)
Alcohol	17	(14%)
Other	18	(14%)
**CRPH**	89	(72%)
**Child-Pugh B–C**	63	(51%)
**Biochemistry**		
Albumin (g l^−1^)	36	(21–55)
PT (%)	70	(31–109)
Bilirubin (µmol l l^−1^)	21	(6–341)
Creatinine (mmol l l^−1^)	84	(50–184)
AST (U/l)	76	(17–492)
ALT (U/l)	59	(10–469)
PST≥1	22	(18%)
**Tumour characteristics**		
Nodule size (cm)	3,5	(0,8–18)
Multinodular	72	(58%)
Gross Vascular Invasion	31	(25%)
**Tumour grading**		
Well differentiated	47	(38%)
Moderately differentiated	52	(42%)
Poorly differentiated	25	(20%)
**UNOS- TNM**		
I–II	60	(48%)
III	26	(21%)
IV	38	(31%)
**BCLC**		
A	58	(47%)
B	30	(24%)
C–D	36	(29%)
**AFP mRNA positive**	50	(40%)
**VEGF>23 pg ml^−1^ (positive)**	44	(35%)
**Therapy**		
Resection	46	(37%)
LPT/VLS ablation	6	(5%)
PEI- RF	48	(38%)
TACE	12	(10%)
LT	12	(10%)

The median size of the largest nodule was 3,5 cm (1 cm to 18 cm), and 72 patients (58%) had multi-nodular HCC.

Radiologic diagnosis of gross vascular invasion was made in 31 patients (25%).

All enrolled patients had histological evaluation of percutaneous/laparoscopic biopsies (60 patients) or surgical specimens (64 transplanted or resected patients). Poorly differentiated tumours (G3) were detected in 25 patients (20%). Staging procedures revealed 64 patients (52%) with Tumour, Nodes and Metastasis (TNM) III–IV tumours and 36 patients (29%) with BCLC C–D hepatic disease.

AFP mRNA determinations in blood preoperative samples yielded a positive result in 50 patients (40%). Serum VEGF values were higher than 23 pg ml^−1^ (positive) in 44 patients (35%).

Liver resection was performed on 46 patients: 17 patients had an anatomic resection (37%), the remaining had a wedge resection. 12 patients (10%) had LT. Six (5%) had laparotomic or laparoscopic radiofrequency thermal ablation. 48 (38%) had percutaneous ablation procedures (31 radiofrequency thermal ablation, 17 Percutaneous Alcohol Injection). 12 had TACE (10%).

After the initial therapy described in Table 1, 54 patients (44% of the cohort) had at least one additional therapy during follow-up: 2 patients had a salvage LT, 2 patients had an additional liver resection; the remaining 40 had a percutaneous ablation or TACE. During the study period (2001–20007) none of the patients were treated with systemic therapy.

### Correlation among AFP mRNA, VEGF determinations and clinical-morphological characteristics

Correlations are described in Table 2. AFP mRNA and VEGF determinations were significantly related to nodule size, gross vascular invasion, AFP
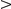
29 ng ml^−1^, TNM and BCLC staging systems, PST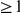, tumour grading, and Child-Pugh B–C stages. Only AFP mRNA positive results were significantly related to Alkaline Phosphatase (ALP) and *γ*-glutamiltransferase (GGT) values and to HCV positive ætiology. Only VEGF determinations were significantly related to PT values and to CRPH.

**Table 2:** Relationship between AFP mRNA/VEGF determinations and clinical-morphological characteristics of enrolled HCC patients.

	AFP mRNA	VEGF
	LR 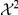 (P)	LR 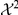 (P)
**Age**>62 years	2–14 (0,1430)	5,07 (0,0243)
**Sex** Male	1,28 (0,2578)	0,13 (0,7211)
**Nodule size**>3,5 cm	2,60 (0,1069)	7,62 (0,0058)
**Multinodular**	0,53 (0,4654)	0,03 (0,8636)
**Gross vascular invasion**	12,91 (0,0003)	15,21 (<0,0001)
**VEGF**>23 pg ml^−1^	18,55 (<0,0001)	—
**AFP mRNA** positive	—	18,55 (<0,0001)
**AFP**>29 ng l^−1^	6,97 (0,0083)	7,27 (0,0070)
**CRPH**	0,13 (0,7182)	5,41 (0,0200)
**Bilobar**	2,94 (0,0865)	1,29 (0,2565)
**Surgery**	0,19 (0,6619)	0,07 (0,7898)
**Albumin**<36 g l^−1^	0,31 (0,5783)	0,09 (0,7635)
**PT**<70%	5,23 (0,0215)	2,40 (0,1209)
**Bilirubin**>21 µmol l^−1^	4,20 (0,0404)	2,99 (0,0839)
**Creatinine**>84 µmol l^−1^	0,16 (0,6869)	0,68 (0,4097)
**AST**>76 U/l	0,65 (0,4201)	0,04 (0,8356)
**ALT**>59 U/l	1,11 (0,2917))	1,72 (0,1891
**ALP**>123 U/l	1,70 (0,1927)	0,06 (0,8011)
**GGT**>68 U/l	3,29 (0,0695)	0,09 (0,7635)
**Child- Pugh** B–C	0,02 (0,8826)	0,02 (0,8940)
**TNM**	18,34 (0,0001)	22,09 (<0,0001)
**BCLC**	13,35 (0,0013)	21,23 (<0,0001)
**PST>1**	8,63 (0,0033)	6,51 (0,0107)
**Grading**	58,35 (<0,0001)	13,04 (0,0015)
**HCV+**	4,20 (0,0458)	3,04 (0,0812)

LR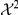, likelihood ratio 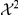 test.

### Univariate survival analysis

The median follow-up of the 124 enrolled HCC patients was 18,6months (range, 1 to 76). During the follow-up, 65 patients (52%) died: 36 from tumour progression, 20 from cirrhosis-related complications, and 9 from non-hepatic-related deaths. Median survival of the study group was 26,4months (range, 1 to 76) and 1, 3, and 5 year survival rates were 71, 46%, and 41%, respectively.

Upon univariate survival analysis (Cox model), the following variables (Table 3) significantly impacted survival in the study group: nodule size 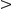
3,5 cm, multi-nodular HCC, gross vascular invasion, grading, AFP mRNA positivity, VEGF
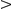
23 pg ml^−1^, Child-Pugh B–C, bi-lobar tumour, surgery, PST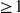, TNM, and BCLC. The integrated AFP mRNA/VEGF variable was considered in two ways. AFP mRNA/VEGF was classified in 3 stages as both negative (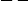), one positive (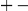 or 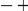), both positive (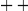). AFP mRNA/VEGF was also classified in 4 stages as both negative (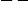), AFP mRNA negative (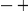), VEGF negative (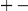), both positive (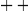).

**Table 3:** Univariate survival analysis: significant survival predictors in the study group.

Variable	Hazard Ratio	
	(95% CI)	*p* Value
Nodule size>3,5 cm	1,28 (1,00–1,65)	0,0461
Multinodular	1,31 (1,01–1,70)	0,0373
Macroscopic vascular invasion	4,01 (2,42–6,57)	<0,0001
**Grading**		
Moderately versus well differentiated	2,88 (1,53–5,73)	<0,0001
Poorly versus moderately differentiated	2,40 (1,36–4,16)	
AFP mRNA positive	3,22 (1,97–5,33)	<0,0001
VEGF>23 pg ml^−1^	2,37 (1,45–3,87)	0,0007
**AFP mRNA/VEGF (3 stages)**		
− + or + − versus − −	2,21 (1,20–4,08)	<0,0001
+ + versus − + or + −	2,31 (1,28–4,20)	
**AFP mRNA/VEGF (4 stages)**		
−+ versus − −	1,78 (0,74–3,90)	<0,0001
+− versus −+	1,56 (0,69–3,85)	
++ versus +−	1,79 (0,94–3,50)	
Bilobar	1,74 (1,01–2,92)	0,0479
Child-Pugh B–C	1,40 (1,09–1,80)	0,0076
Surgery	1,64 (1,28–2,13)	0,0001
PST>1	4,12 (2,37–6,93)	<0,0001
**TNM**		
III versus I–II	1,91 (0,94–3,78)	<0,0001
IV versus III	2,55 (1,38–4,97)	
**BCLC**		
B versus A	2,04 (1,00–4,15)	<0,0001
C–D versus B	3,52 (1,94–6,71)	

AFP mRNA/VEGF (3 stages): stage 1 = − −, stage 2 = − + or + −, stage 3 = + +.

AFP mRNA/VEGF (4 stages): stage 1 = − −, stage 2 = − +, stage 3 = + −, stage 4 = + +.

**Figure 1:**
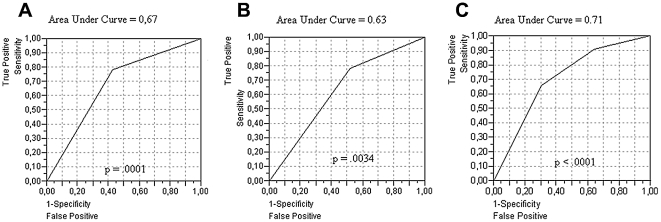
ROC curve analysis. Relationship between patient mortality and AFP mRNA used alone (A), VEGF used alone (B), and the integrated use of the 2 markers (C).

**Figure 2:**
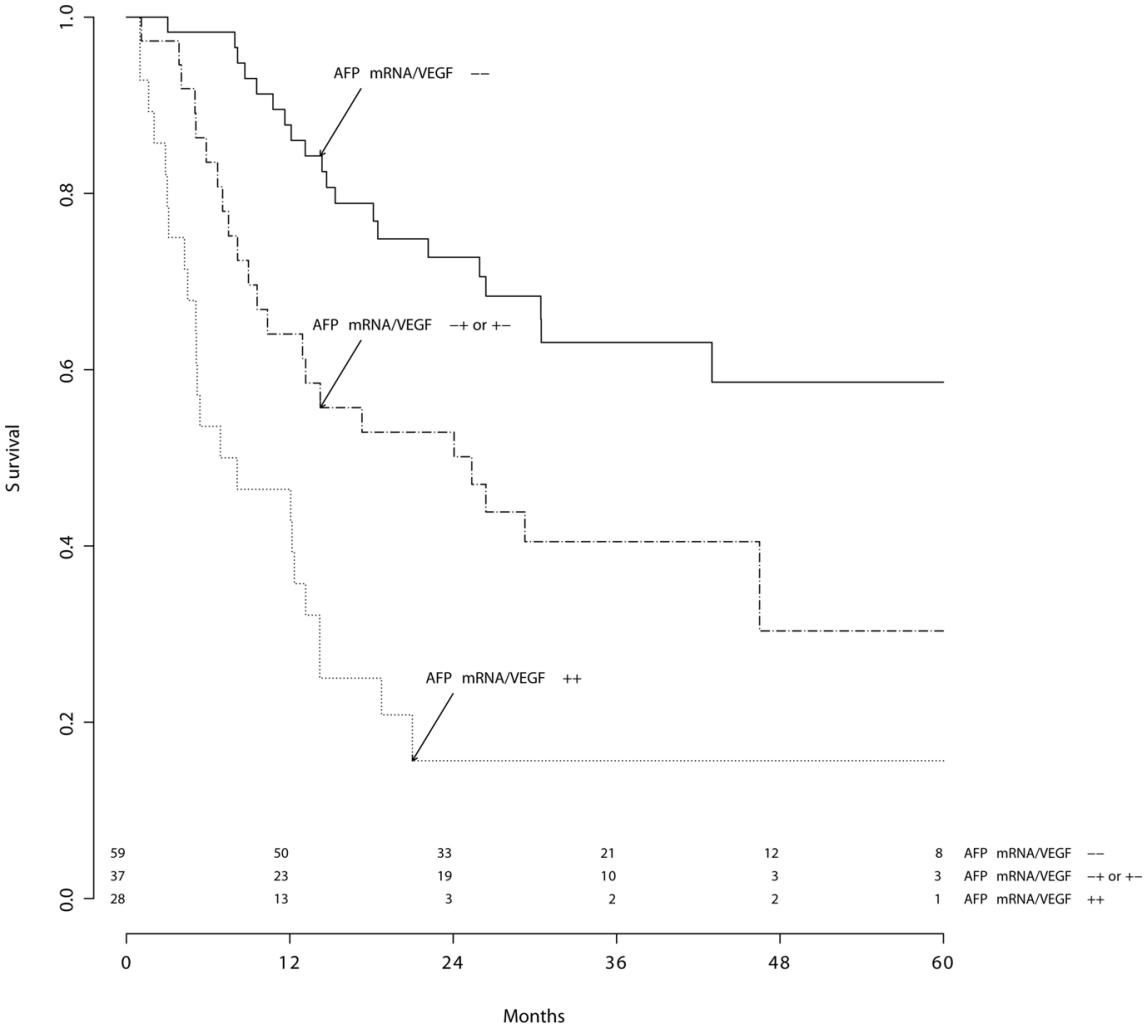
Prognostic classification of the study group according to AFP mRNA/VEGF. Molecular staging of HCC patients: integrated variable (3 stages). Log rank test: 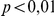.

The Cox model and ROC curve analysis showed that the best interpretation of survival distribution was given by an association of AFP mRNA and VEGF in 3 stages (Figures 1–2).

### Multivariate survival analysis

Upon multivariate analysis (Table 4) of the overall study group, BCLC staging, surgery, gross vascular invasion and the AFP mRNA/VEGF integrated variable maintained a significant impact on survival.

**Table 4:** Evaluation of the independent contribution of each variable to Cox Proportional Hazard Model. Multivariate Survival Analysis using AFP mRNA and VEGF as an integrated variable.

Variable	Hazard Ratio	
	(95% CI)	*p* Value
Nodule size>3,5 cm	1,24 (0,87–1,78)	0,2389
Multinodular	1,28 (0,92–1,82)	0,1481
Macroscopic vascular invasion	0,35 (0,12–1,01)	0,0517
**Grading**		
Moderately versus well differentiated	1,76 (0,84–3,81)	0,1049
Poorly versus moderately differentiated	1,54 (0,74–3,25)	
**AFP mRNA/VEGF (3 stages)**		
− + or +− versus − −	2,36 (1,14–4,99)	0,0305
+ + versus − + or + −	1,12 (0,52–2,41)	
Bilobar	0,72 (0,36–1,40)	0,3360
Child- Pugh B–C	1,26 (0,93–1,74)	0,1379
Surgery	1,66 (1,22–2,30)	0,0011
PST>1	1,33 (0,64–2,72)	0,4351
**TNM**		
III versus I–II	1,58 (0,60–4,07)	0,6306
IV versus III	0,89 (0,38–2,15)	
**BCLC**		
B versus A	0,74 (0,29–1,91)	0,0055
C–D versus B	6,30 (2,08–18,53)	

AFP mRNA/VEGF (3 stages): stage 1 = − −, stage 2 = − + or + −, stage 3 = + +.

AFP mRNA/VEGF (4 stages): stage 1 = − −, stage 2 = − +, stage 3 = + −, stage 4 = + +.

### Molecular staging of homogeneous subgroups of HCC patients

The AFP mRNA/VEGF integrated variable was also tested in the following homogeneous subgroups of HCC patients: BCLC A patients, BCLC B patients and patients treated by resection or ablation procedures with a curative intent (BCLC A–B patients).

**Figure 3:**
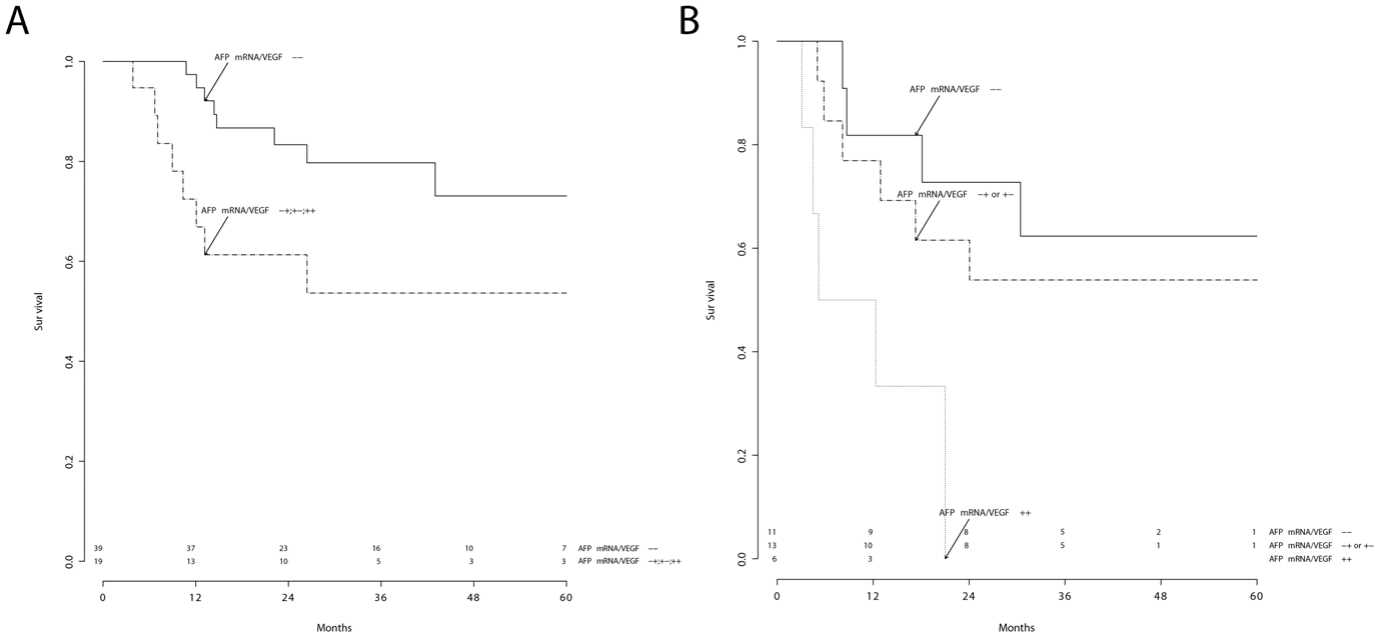
Molecular staging of HCC patients. Comparison between BCLC A (A) and BCLC B (B) stages. Log rank test: 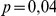 (A), and 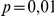 (B).

**Figure 4:**
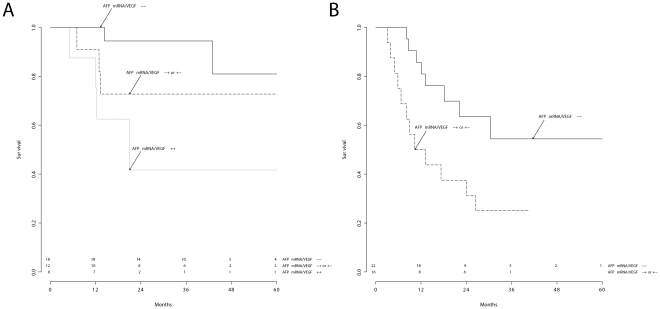
Molecular staging of surgically treated BCLC A–B HCC patients. Patients treated by resection (A) or ablation procedures (B).

The proposed molecular staging showed significant discriminatory ability (log-rank test applied to Kaplan-Meier analysis) in BCLC A and B patients (Figure 3, A–B) but not in BCLC C–D patients (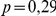). The AFP mRNA/VEGF integrated variable also showed significant prognostic value in patients undergoing resection (Figure 4A) and ablation (Figure 4B).

## Discussion

This prospective observational study was specifically designed to evaluate the prognostic role of the preoperative determination of VEGF serum levels and AFP mRNA in a cohort of HCC patients in order to evaluate their indication for potentially curative therapies.

The results of the present study should therefore be interpreted by considering the potential utility of these still experimental molecular markers in daily clinical practise.

Prognostic factors may help the treatment strategy for HCC patients in different ways:

1. in deciding the best therapy according to patient characteristics;
2. in estimating individual survival perspectives;
3. in designing clinical trials to test new therapies;
4. in programming post-treatment follow-up and adjuvant therapy;

Prognostic prediction and therapeutic decisions for HCC patients in most centres are currently based on macroscopic tumour characteristics detected by imaging studies (nodule size and number). In the past decade, the empirical rule of a single nodule smaller than 5 cm in diameter or multiple nodules (2 or 3) smaller than 3 cm has been used to define early HCC, with excellent outcomes achieved after LT [6], [13].

These macro-morphological tumour characteristics have been incorporated in the BCLC classification [14] to discriminate between early and intermediate/advanced stages. Moreover, according to this staging system, advanced or terminal stages may also be identified by severe liver impairment (Child-Pugh C) or compromised performance independently from tumour stage, because these characteristics may either limit the individual therapeutic success or indirectly identify more aggressive tumours.

BCLC staging is now considered the best available prognostic system for HCC patients [6], [7], and our centre has made an important contribution to the validation of this staging system [15], [16], particularly in populations mainly characterized by radically treated HCC patients.

Therefore, we agree that BCLC clinical variables (radiological tumour size, number and vascular invasion; liver function parameters; performance status) are crucial prognostic factors for patients with HCC. In addition, in this prospective study, these variables demonstrated significant discrimination of survival ability (Table 3) on univariate analysis.

However, there is a substantial variation in prognosis among patients within the same BCLC stage [7]. This fundamental concept has been confirmed in the present study, which showed that molecular staging had significant predictive ability on multivariate analysis, together with surgery and BCLC staging.

The first result of this study, therefore, is that our proposed molecular staging may potentially refine the prognostic evaluation of patients with HCC. Circulating prognostic molecular markers (AFP mRNA and VEGF) showed significant ability in discriminating survival within BCLC stages, especially when used as an integrated variable (Figure 3).

The prognostic heterogeneity makes BCLC staging alone relatively inaccurate for determining the best treatment strategy and estimating individual prognosis. As shown in recent studies [17], [18], there is a substantial therapeutic hierarchy within each BCLC stage that is prognostically characterized by surgery as the first therapeutic option, percutaneous ablation as the second, TACE as the third, and, finally, systemic therapies as the fourth. This evidence was also confirmed in the present analysis by multivariate analysis, which showed the prognostic independence of surgical therapy with respect to BCLC staging (Table 4).

Thus, in therapeutic decisions, negative prognostic factors do not necessarily coincide with absolute exclusion criteria for a specific therapy. For example, multi-nodularity, vascular invasion and portal hypertension [19], [20] in Child-Pugh A patients are negative prognostic factor for patients undergoing liver resection but the therapeutic alternatives in these subgroups of patients are often very poor. In this view, the proposed BCLC treatment algorithm [7] may not be used to formulate strict therapeutic guidelines [6].

The small sample size in this study does not allow us to reach definitive conclusions about the potential role of these molecular markers in treatment decision. On a purely speculative basis, the results of this study suggest the potential utility of these biomarkers in excluding potentially curative therapies for BCLC B patients with a poor prognosis (those with a simultaneously positive blood value for AFP mRNA and VEGF, Figure 3B).

As a third point, these prognostic factors should help design RCT stratifying the overall HCC populations in prognostically homogeneous subgroups. In this context, the BCLC system has the relevant merit of favouring the development of RCT, especially in the complex field of HCC treatment assessment [21], [22]. From these perspectives, molecular markers probably do not add much to this particular clinical and scientific field.

However, post-treatment follow-up and adjuvant therapy concerns remain a crucial and non-standardized problem. The majority of BCLC A patients will develop a tumour recurrence within 5years from treatment, despite undergoing potentially curative therapies such as resection or ablation [23]. Moreover, to extend the indication of potentially curative therapies to BCLC B patients as well, it would be important to reduce the risk of post-treatment recurrence or to make an early diagnosis.

In this view, molecular markers capable of discriminating the prognosis of patients within BCLC stages could be important for post-treatment follow-up and preventive therapy.

The second fundamental result of this study is that blood AFP mRNA and VEGF showed a relevant prognostic ability in BCLC A–B patients (Figure 3, A & B), particularly in selected subgroups undergoing resection or ablation (Figure 4, A & B). Although this study was not specifically designed to study post-treatment recurrence, our results suggest that patients with one or two pre-operatively positive molecular markers should be strictly monitored and could eventually undergo adjuvant therapies in order to improve their post-treatment prognosis.

Their strong correlation with tumour grade and vascular invasion (Table 2) and their prognostic power (Table 3, Table 4) suggest that blood AFP mRNA and VEGF are direct markers of biological tumour aggressiveness.

In light of the recent introduction of molecular targeted therapies [24] acting on pathways of tumour biological progression, it is possible that our proposed molecular staging might help to select HCC patients with higher potential for good response to these new therapies. In particular, this may be true for Sorafenib [22], which mainly acts by inhibiting VEGF cellular signalling implicated in HCC pathogenesis and progression.

Previous experiences exploring the prognostic role of preoperative AFP mRNA and VEGF have yielded controversial results [8], [9], [12]. It is likely that these biologic HCC parameters alone do not reach adequate prognostic power in highly selected populations undergoing potentially curative therapies. The present study suggests that in the context of a larger population basis, AFP mRNA and VEGF may be useful for both prognostic assessment and treatment decisions, especially when used as an integrated variable. The introduction of appropriate molecular methods for quantifying AFP mRNA in the blood and larger studies to identify the exact prognostic range of VEGF will further improve the prognostic power of these promising biomarkers.

In conclusion, this study has shown that preoperative determination of blood AFP mRNA and VEGF may potentially refine the prognostic evaluation of HCC patients. On a purely preliminary basis, these two parameters may help in the treatment decision process for HCC patients eligible for potentially curative therapies, consequently improving the currently used restrictive selection criteria.

## Methods

### Ethics Statement

In the period the study was realized (2001–2007) the Ethics Committee of our institution at the University Hospital of Padua did not specifically require an explicit approval for anonymized prospective observational studies. Patients were informed about the research, but no informed consent was requested since all data was anonymized.

### Study design

This prospective observational study started in January 2001. Inclusion criteria were: (a) histological diagnosis of HCC and assessment of tumour grade (percutaneous biopsy or histology of surgical specimens); (b) baseline determination of AFP mRNA status and VEGF levels in the blood; and (c) a general consent for the use of a blood sample for research purposes. Exclusion criteria were: (a) fibrolamellar HCC; (b) concomitant extra-hepatic tumour; (c) HIV positivity; and (d) previous recent HCC treatment (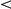
6months).

At the moment of patient enrolment and baseline evaluation, we recorded the following variables: age, sex, and cirrhosis ætiology; PST, presence of CRPH defined as the presence of gastro-œsophageal varices, splenomegaly with a platelet count of less than 100000 cells/ml, ascites; main serological parameters [total bilirubin, AST, ALT, PT, albumin, ALP, GGT, creatinin]; Child-Pugh class; and tumour characteristics [number and size of lesions, gross vascular invasion, metastasis, AFP levels, UNOS-TNM (TNM) [13], and BCLC staging [14]. Microscopic vascular invasion could only be determined in a minority of cases due to lack of a surgical specimen, thus this variable could not be studied. Blood samples for AFP mRNA and VEGF determinations were taken 2 h to 48 h before therapy. The minimum follow-up for all patients included a clinical visit, hepatic US, and blood analysis with AFP levels every 3 months in the first year and every 6 months in the second and third years.

### AFP mRNA determination

An EDTA-K3-supplemented blood sample (7 ml) was obtained from all subjects and immediately stored at 4°C for no more than 3 h until processing. Total RNA was extracted and converted to cDNA. A nested polymerase chain reaction protocol was performed as previously reported [10]. Positive results were observed as a 282 bp band in an agarose gel stained with ethidium bromide. As positive control, cDNA obtained from the hepatocellular carcinoma cell line HepG2 was used in each run. Negative controls for each step were also included. The sensitivity of AFP mRNA determination was 10 cells in 7 ml of blood and measured by diluting HepG2 cells in blood samples obtained from negative subjects.

### Serum determinations

A serum sample was also obtained from each subject and stored at −20°C until use in AFP protein and VEGF immunoassays (Bayer, Milan, Italy, and BioSource International, Nivelles, Belgium, respectively). The sensitivity of the assay for VEGF was 23 pg ml^−1^, which was the minimum VEGF concentration with a coefficient of variation of less than 20%. The intra-assay precision for VEGF was less than 5% in the concentration range 87 pg ml^−1^ to 938 pg ml^−1^. The intra-assay precision for AFP protein was less than 5% in the range 20 ng ml^−1^ to 732 ng ml^−1^.

### Plasma determination

All patients were also tested for albumin, bilirubin, creatinin, AST, ALT, ALP, GGT and PT using standard methods.

### Treatment algorithm

**Figure 5:**
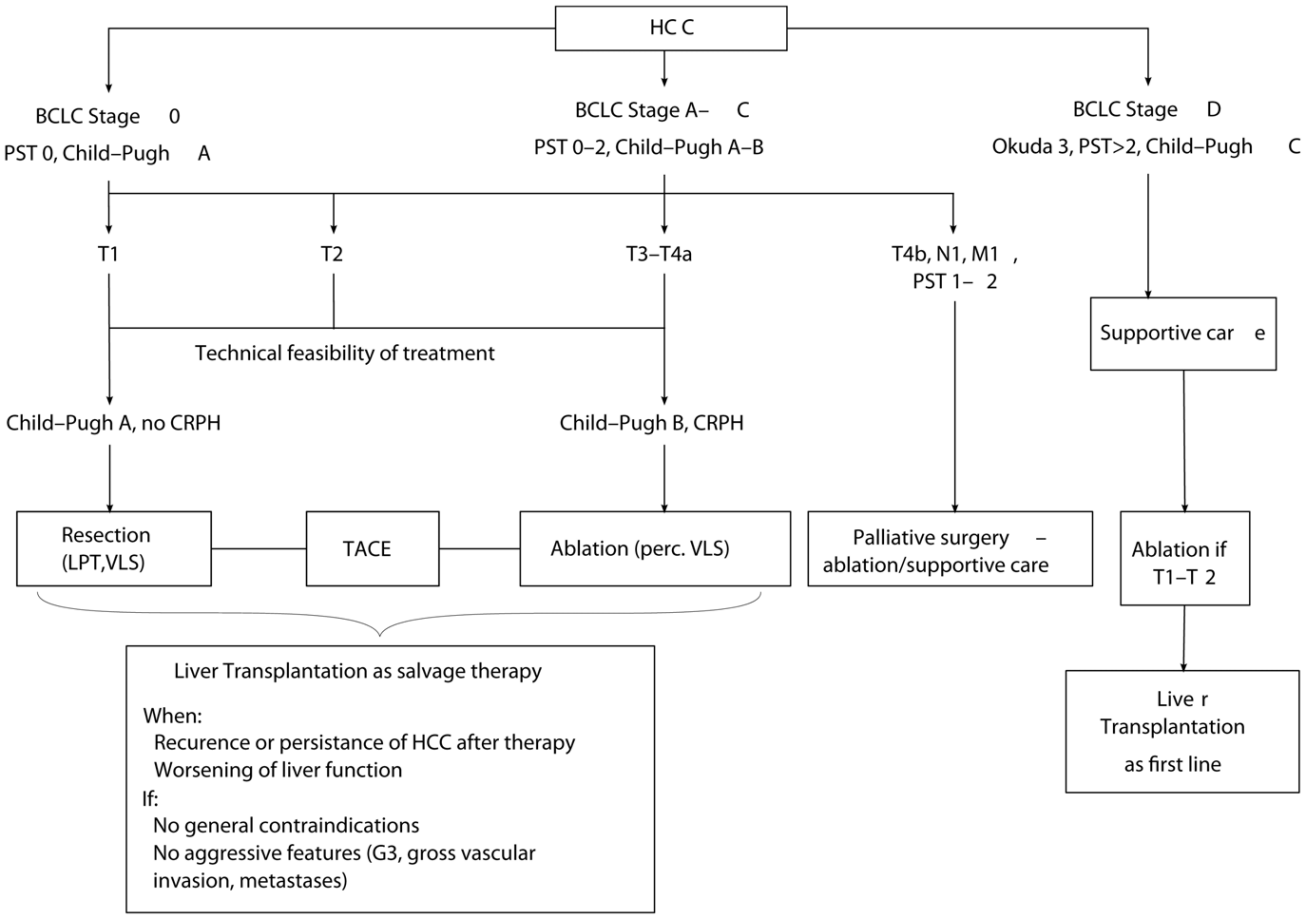
Treatment algorithm.

All enrolled patients were referred to our tertiary hepatobiliary surgery-liver transplantation unit to evaluate the feasibility of potentially curative therapies. Patients in BCLC A–B stages were considered for surgery or ablation procedures eventually associated with TACE: as previously reported [25], the rate of anticancer therapeutic aggressiveness in these patients was determined at our institution mainly by liver function parameters such as Child-Pugh class, CRPH and, in the last 4years, the MELD score, whereas nodule size and number were not absolute criteria for assigning potentially curative treatment (Figure 5). LT was considered a salvage treatment for all patients having HCC persistence/progression or recurrence or having severe liver function impairment (BCLC D). Absolute exclusion criteria for LT were [26]: general contraindications (age, extra-hepatic disease, compliance) or aggressive tumour features (poorly differentiated grade, gross vascular invasion, metastasis).

Patients with advanced tumours (gross vascular invasion, metastasis) but good liver function (BCLC C) were eventually considered for surgery or ablation only from a palliative perspective.

### Aims of the study

1. To investigate the correlation between AFP mRNA and VEGF determinations and clinical-morphological characteristics of enrolled HCC patients.

2. To evaluate the prognostic role of AFP mRNA and VEGF determinations used alone or together as an integrated variable.

### Statistical analysis

The patients' baseline characteristics are expressed as medians (range) for continuous data and as frequencies (percentage) for categorical data. There were only 9 (7%) Child-Pugh C patients; thus, we considered only 2 stages, Child-Pugh A and B–C. Child-Pugh C patients were considered BCLC D only when they were without LT therapeutic perspectives. Because the TNM I stage included only 9 patients (7%) and the BCLC D stage only 4 patients (3%), the following variables were stratified in three main stages: BCLC A, B, C–D and TNM I–II, III, IV, respectively. Because only 44 patients (35%) had a serum VEGF value higher than 23 pg ml^−1^ (sensitivity of the assay), this variable was considered a categorical variable identifying the 2 subgroups of patients with VEGF values
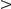
23 pg ml^−1^ (positive) or 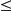
23 pg ml^−1^ (negative). Comparisons between groups were analyzed by logistic regression, the 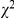 test or Fisher's exact test, as appropriate. Follow-up and survival times are expressed as medians (range). Survival was calculated from the day of AFP mRNA/VEGF determinations until death or latest follow-up. The study period was between January 2001 (first enrolment) and September 2007 (last follow-up).

Cox's univariate proportional hazards models were used to identify potential survival predictors in the study group. Cox regression and ROC curve analysis were used to evaluate the interaction between AFP mRNA and VEGF determinations and to determine the prognostic utility of a potential integrated variable, taking into account both molecular markers.

A Cox's multivariate model was then created, including only variables with 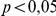 at univariate analysis.

Probability curves for survival were calculated using the Kaplan-Meier method, and the log-rank test was applied to the Kaplan-Meier curves to identify potential predictors of survival.

In order to reduce the confounding effect of treatment and staging on survival response variables, the prognostic impact of the molecular markers was also tested in subgroups of BCLC A, BCLC B, and BCLC A–B patients undergoing resection or ablation.

Statistical significance was set at 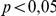, and calculations were performed with the JMP package (1989 to 2003 SAS Institute Inc.).
